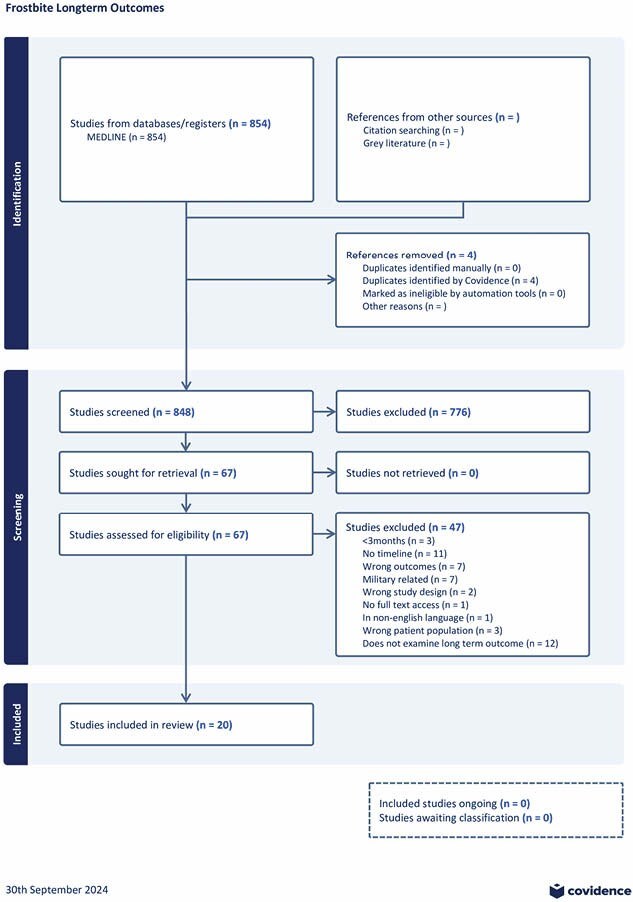# 905 Long Term Outcomes of Frostbite: A Scoping Review

**DOI:** 10.1093/jbcr/iraf019.436

**Published:** 2025-04-01

**Authors:** Abby Rentz, Sarvesh Logsetty, Rae Spiwak, Justin Gawaziuk, Gursagar Jhanji, Brenda Comaskey

**Affiliations:** Manitoba Firefighters Burn Unit; Manitoba Firefighters Burn Unit; University of Manitoba; University of Manitoba; University of Manitoba; University of Manitoba

## Abstract

**Introduction:**

Frostbite injury is an important cause of morbidity in cold climates. The initial clinical presentation may not correlate with the final outcome; some injuries completely resolve while others lead to tissue loss or amputation. Studies have identified sequelae of frostbite which persist beyond the acute injury phase, such as pain, stiffness and cold intolerance. The body of evidence surrounding long term outcomes (LTO) of frostbite is limited, consisting mostly of small studies and case reports. The aim of this scoping review was to further characterize LTO in frostbite injury.

**Methods:**

The scoping review process was guided by the methodology framework of Arksey and O’Malley and the Preferred Reporting Items for Systematic Reviews and Meta-analysis Protocols Extension for Scoping Reviews Guidelines (PRISMA-ScR). MedLine database search was performed to identify studies between January 1, 2000 and May 8, 2023 that examined LTO (persisting 3 months post-frostbite injury) in adults aged 18 or older. All articles identified from the database search were screened by two independent reviewers and included if eligibility criteria were met. Duplicate articles were removed, and full texts were examined to create a final list of included studies. Any disagreements on the inclusion of articles were resolved by a third reviewer.

**Results:**

The database search identified a total of 850 studies and 74 were retrieved for further evaluation, of which 20 were retained in the final review. There were 10 case reports, 4 retrospective reviews, 2 prospective studies, 2 case series, 1 scoping review, and 1 book chapter. Of the included studies, 17 attributed specific long term outcomes to individuals. In 13 of 17 studies, long term sequelae of frostbite were reported in 395/624 patients (63.3%). Commonly cited physical sequelae included chronic pain, frostbite arthritis, stiffness/reduced dexterity, cold hypersensitivity, and reduced sensation. Psychosocial sequelae included poor emotional well-being, social limitations, and lowered working ability secondary to injury.

**Conclusions:**

The experience of adverse LTO following frostbite injury is common amongst survivors. The results from this review highlight a significant gap in the understanding of the etiology, incidence and impact of chronic sequelae following frostbite injury.

**Applicability of Research to Practice:**

Further study is required to determine how to counsel and support affected individuals.

**Funding for the Study:**

This research is supported by a Canadian Institutes of Health Research Catalyst Grant. The funding source had no role in the design and development of the protocol, nor in the conduct of the study including collection, management, analysis and interpretation of data and preparation, review and approval of the manuscript.